# Relationship between Presentation, Attitude, and In-Flight Meal Food Healthiness: Moderating Role of Familiarity

**DOI:** 10.3390/foods13132111

**Published:** 2024-07-02

**Authors:** Ryeojin Jang, Won Seok Lee, Joonho Moon

**Affiliations:** 1 Department of Tourism and Recreation, Kyonggi University, Suwon 443760, Republic of Korea; flyjin2@daum.net (R.J.); lws79877@gmail.com (W.S.L.); 2 Department of Tourism Administration, Kangwon National University, Chuncheon 24341, Republic of Korea

**Keywords:** in-flight meal, food healthiness, presentation, attitude, familiarity

## Abstract

This work focused on the perception of the food healthiness of in-flight meals. This work adopts presentation as the determinant. This work also employs attitude as the consequence of food healthiness. This research also examines the moderating effect of familiarity on the relationship between food presentation and food healthiness. This research used a survey, and survey participants were recruited via a Clickworker platform service. Survey participants were experienced with in-flight meals. The number of observations was 317. Moreover, this research tested the research hypotheses using the Hayes process macro Model 7. The results revealed that food healthiness is positively influenced by presentation and that food healthiness positively affects attitude. Moreover, the results revealed that the type of presentation has a positive influence on attitude. Familiarity was a significant moderating variable for the relationship between food presentation and food healthiness. This work sheds light on the literature by identifying the associations among four attributes of in-flight meals. Additionally, the results of this study could be used as a reference to develop better in-flight meals.

## 1. Introduction

In-flight meals are a crucial element of airline service. Moreover, scholars have shown that in-flight meal consumption is associated with seat quality, which implies that in-flight meals are consumed more in uncomfortable situations [1,2]. Previous research has found that food healthiness is important in the area of in-flight meals because food needs to be digested well and make passengers feel less stressed during hard and boring travel, as well as offer sufficient nutrition in an airplane [3,4,5,6]. Indeed, scholars have claimed that food healthiness is an important issue for in-flight meals because healthy food becomes a source of energy for long stays on airplanes [4,7]. By integrating the arguments of previous works, it can be inferred that the food healthiness of in-flight meals is likely to become an essential area to investigate because healthy food is useful for making passengers more energetic and less stressed over a long journey.

The central attribute of this work is food healthiness. The extant literature has shown that food healthiness has become a more important trend in the food service market because individuals have become more interested in their health and diet, which are both impacted by food consumption [5,6,8]. Vera-Santander et al. [9] defined food healthiness as food that offers nutrition which promotes health, and Plasek et al. [6] noted that food healthiness is associated with the consumption of nutritional food. Focusing on the arguments of the prior literature, this research defines food healthiness as the consumption of nutritional food for better health conditions. Moreover, Han et al. [4] performed empirical research to examine the consumer perception of in-flight meals using food healthiness as the main attribute. Integrating the extant literature, this study adopts food healthiness as the main variable. In addition, attitude is a dependent variable in this study. Attitude has been commonly explored by scholars because it reflects the long-term appraisal of individuals [10,11]. In addition, Xie et al. [12] stated that attitudes toward food differ according to individual background and situation. This finding implied that food healthiness is likely to be unique in the case of in-flight meals because in-flight meal consumers have varied cultural backgrounds and are experiencing discomfort, such as narrow seats and high altitudes. This suggests that it is worthwhile to inspect the determinants of attitudes toward in-flight meals.

This work selects food presentation as the explanatory variable. Researchers contend that presentation is the first impression of food [13,14]. Previous research has shown that food presentation accounts for the appraisal of consumers because of visual stimuli [6,15]. Moreover, prior works have shown that food presentation is likely to serve as a cue for how food promotes or degrades one’s health because it enables consumers to guess ingredients and freshness [16,17]. The last domain of this work is familiarity. Individuals dislike uncertainty in their decision-making, and this finding can be applied to in-flight meals. Because airline passengers have varied cultural backgrounds toward food, familiarity is likely to work as a critical attribute to explain consumer behavior. Hagen [18] suggested that consumer reactions to food are likely to differ depending on familiarity because food consumption is strongly influenced by cultural background and experience. It can be inferred that familiarity is likely to work as a moderating variable to explain food consumer behavior. Thus, this work investigated the moderating impact of familiarity on consumer behavior in the context of in-flight meals.

Overall, the purpose of this work is to examine the antecedents and consequences of food healthiness for in-flight meals. Attitude is likely to become a consequence of food healthiness, while food presentation is a determinant of food healthiness. The hypothesis was implemented based on the findings and argument of the extant literature. Another goal of this work is to investigate the moderating effect of food familiarity. To be specific, the independent variable of this work is presentation, while food healthiness is the mediator. Additionally, attitude is the dependent variable, and familiarity is the moderating variable for the effect of presentation on food healthiness. This research contributes to the literature by identifying the relationships among four attributes. Because scant studies have been implemented to figure out consumer behavior in the area of in-flight meals, this work sheds light on the literature by streamlining such a research gap. In addition, this work presents information to improve the quality of in-flight meals from the perspective of consumers.

## 2. Literature Review and Hypothesis Development

### 2.1. Attitude

An attitude is a settled appraisal of a certain target [19,20]. Many studies have used attitude as their main attribute. For instance, Wong et al. [21] explored the perception of renewable energy users using attitude as the main attribute. Bunz et al. [20] used attitude as an explanatory variable to explore virtual reality technology users. Natasia et al. [22] investigated the influence of attributes on attitude in e-learning. Loera et al. [23] employed attitude in research on organic food consumers. Gansser and Reich [24] chose attitude as a central element to explore behavior towards the environment. Liao et al. [25] explored the influence of attitude toward low-carbon travel. In a similar vein, Zhao et al. [11] scrutinized the influence of attitude in the domain of agro-tourism. Focusing on food research, Baiano [26] explored the influence of attitude in the case of 3D-printed food. Desye et al. [27] also researched the link between food safety and attitude. Roy et al. [28] additionally researched consumer attitudes toward organic food empirically. Plus, Hussain et al. [29] used attitude as an explanatory variable to explore the local food consumer value effect. In sum, many studies have been performed using attitude as the explanatory attribute.

### 2.2. Food Presentation

Presentation is defined as the visual aspect of food [13,14]. Plasek et al. [6] reported that sensory attributes are essential determinants of the perception of food healthiness through a structured review of the literature. Verma [14] reported that food presentation is essential for marketing in the domain of food delivery application systems because consumers like to see pictures. Namkung and Jang [14] also showed the positive impact of food presentation on customer satisfaction. Overall, visually appealing food is likely to drive positive assessments of food.

### 2.3. Food Healthiness

Food healthiness refers to how food is useful for promoting individual health [5,6]. Petrescu et al. [5] reported that consumers assess food healthiness using clues such as the freshness of ingredients, nutritional facts, proportion of fruit and vegetables, and additives. Many studies have examined food healthiness. Plasek et al. [6] reported that food healthiness is key for sustainability because food consumption patterns are closely linked with individual health conditions. Hosni et al. [8] and Vera-Santander et al. [9] reported that the market values healthy food more because healthy food brings about a better quality of life. Hallez et al. [30] stated that food healthiness can be used as a persuasive marketing tool on packaging labels. Rana and Paul [31] found that interest in food healthiness is an imperative motivation for consuming organic food.

### 2.4. Hypothesis Development

Amar et al. [32] claimed that individuals use visual signals to appraise whether food promotes health. Hagen [18] showed that the perception of food healthiness, which emphasizes healthy ingredients, is affected by food presentation. Buhrau and Ozturk [16] and Chan and Zhang [17] reported that food presentation is a critical item for consumers when assessing food healthiness because it provides useful information on ingredients. Lee et al. [33] and Chan and Zhang [17] contended that food presentation plays a significant role in building consumer attitudes because the presentation gives consumers the first impression of the food. Lee et al. [33] indeed found a positive influence of food presentation on attitude. Jang et al. [34] reported that food presentation crucially affects consumers’ reactions to Korean food. Chousou and Mattas [35] revealed a positive impact of food presentation on attitude. Gunden et al. [36] explored users of food delivery applications and found that food presentation using pictures is an essential determinant of attitude. Thus, this work proposes the following research hypotheses:

**Hypothesis** **1.** *Presentation positively affects the perception of the food healthiness of in-flight meals*.

**Hypothesis** **2.** *Presentation positively affects attitudes towards in-flight meals*.

Åstr^⊘^sm and Rise [37] contended that food healthiness is critical for building a positive attitude among consumers. Cuesta-Valiño et al. [38] adopted social marketing for the food market by implementing structural equation model analysis and found a positive effect of perception of food healthiness on attitude. Nystrand and Olsen [39] reported a positive effect of the perception of food healthiness on attitude for functional food consumers in Norway. In a similar vein, Govaerts and Olsen [40] studied seaweed consumers and found that the perception of food healthiness positively impacts attitude. Additionally, Han et al. [4] found a positive effect of the perception of food healthiness on the attitudes of in-flight meal consumers. Therefore, this research proposes the following research hypothesis:

**Hypothesis** **3.** *The perception of the food healthiness of in-flight meals positively affects attitude*.

### 2.5. Familiarity

Previous studies have shown that individuals dislike uncertainty in decision-making, which could become synonymous with risk and unfamiliarity because such aspects lead individuals to feel uncomfortable and nervous [19,41,42]. For instance, Im et al. [41] studied Korean consumers and noted that consumers tended to avoid risk in their decision-making process. This implies that individuals prefer familiar products. Indeed, scholars have revealed the importance of consumer familiarity for the food industry. In fact, Wang et al. [19] claimed that minimizing uncertainty in the selling process is important for positive appraisal. In the food research literature, Tuorila and Hartmann [43] suggested that unfamiliar food causes negative consequences such as disgust and neophobia because unfamiliar food is likely to result in negative consequences: food poisoning and allergies. Jang and Kim [44] reported that food familiarity significantly affects consumer evaluation in the case of ethnic food. Hagen [18] contended that aesthetic food is perceived as healthy when it is presented in a safer manner. Safety is likely to be interpreted as familiarity regarding the degree of uncertainty from the viewpoint of the consumer. Hence, it can be inferred that in-flight meals are likely to be perceived as healthier if the food is familiar and has a visually appealing presentation. Hence, this research proposes the following research hypothesis:

**Hypothesis** **4.** *Familiarity significantly moderates the effect of presentation on the perception of the food healthiness of in-flight meals*.

## 3. Method

### 3.1. Research Model

**Figure 1:**
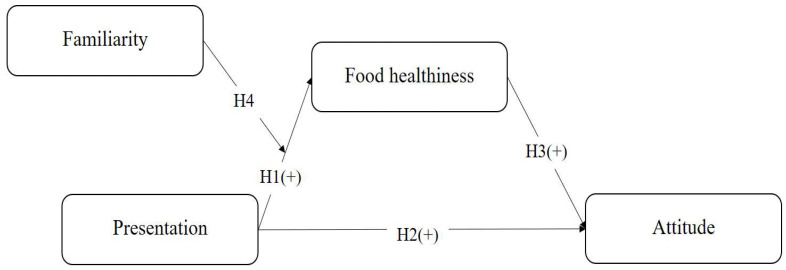
Research model.

Figure 1 shows the research model. There are four variables in this work: presentation, perception of food healthiness, attitude, and familiarity. Presentation positively impacts both the perception of food healthiness and attitude. The perception of food healthiness is positively associated with attitude. Moreover, familiarity significantly moderates the relationship between the presentation and perception of food healthiness.

### 3.2. Illustration of Measurement Items

This research used a five-point Likert scale for the measurement of presentation, healthiness, and familiarity, and a semantic differential scale (e.g., 1 = bad, 5 = good) to measure attitude. This research referenced the extant literature for the measurement of perceptions of food healthiness [45,46]. This research also derived items from previous research for presentation [47,48] and attitude [49,50]. After the derivation, this research adjusted the measurement times to become more suitable for the purpose of the study. Additionally, this research consulted experts in tourism research to develop familiarity measurement items. Considering the operational definitions of these attributes, the perception of food healthiness is defined as how in-flight meals are perceived as being useful for improving consumer health, focusing on nutritional value. The operational definition of attitude is a long-term appraisal of an in-flight meal. Presentation is defined as how respondents perceive the visual aspect of an in-flight meal in this work. Last, the definition of familiarity is how familiar the in-flight meal was to the survey participants in this research.

### 3.3. Data Collection and Analysis Procedure

This research recruited survey participants using a Clickworker system (https://www.clickworker.com/, accessed on 30 January 2024) for data collection. Many studies have used Clickworkers for data collection, which enables researchers to guess that data quality is likely to be assured in Clickworker systems [51,52]. The targets of the data collection were native English speakers in the US, and survey participants had to be older than 20 years old. The data collection period was between 1 February and 7 February in 2024. First, 364 observations were collected, and 47 observations were eliminated because they had no experience with in-flight meals. Namely, this study used a screening question at the beginning of the survey to determine whether the participants were experienced with in-flight meals or not because it aimed to collect data based on the vivid experiences of customers to ensure the better quality of the data. Plus, this research provided no photos to the survey participants because in-flight meal quality might vary depending on airlines. Thus, this research used 317 observations for data analysis using convenience sampling, which collects market research data from a conveniently available pool of respondents randomly [53] because convenience and random sampling are likely to reflect the market condition well, as well as lower the likelihood of bias in the estimation [53].

This research implemented frequency analysis for demographic information. This work also performed exploratory factor analysis with varimax rotation to ensure the validity of the measurement items using the following criteria: the loading threshold was 0.5, the eigenvalue was 1, the Kaiser–Meyer–Olkin (KMO) measure of sampling adequacy was 0.7, and the statistical significance of Bartlett’s test of sphericity (χ^2^) had a 95% confidence interval [53]. This study chose a Cronbach’s alpha of 0.7 to test the reliability of the measurement items. Additionally, this study calculated the mean and standard deviation (SD) of the main attributes; this work also used a correlation matrix to determine the associations between elements. Furthermore, this work used the ordinary least squares-based Hayes process macro Model 7 with 5000 bootstrap replicates as an instrument for hypothesis testing. Estimation using the Hayes process model is less likely to be undermined by bias because the normality assumption is less strict [54]. This research additionally implemented median split analysis to inspect the moderating effect of familiarity.

## 4. Results

### 4.1. Profile of Survey Participants

Table 1 displays the demographic information of the respondents from the convenience and random sampling method. The percentages of males and females were 19.2% and 80.8%, respectively. The age information is depicted in Table 1 (in their 20s: 80; 30s: 127; 40s: 83; 50s: 21, and older than 60: 6). The monthly household income information is presented in Table 1 (under USD 2500: 55 (17.4%), USD 2500–4999: 93 (29.3%), USD 5000–7999: 65 (20.5%), USD 7500–9999: 36 (11.4%), and over USD 10,000: 68 (21.5%). Regarding the annual use frequency of in-flight meal services, 1–2 times accounted for the largest proportion (184, 58.0%).

### 4.2. Results of Exploratory Factor Analysis and Reliability Test

Table 2 shows the validity and reliability of the measurement items. Considering the KMO statistics and Bartlett’s test of sphericity χ^2^ (*p* < 0.05), the model is statistically significant. All factor loadings are greater than 0.5, and all values of Cronbach’s α are greater than 0.7. The results indicated that the validity and reliability of the scale are acceptable. As a result, four variables are derived from the results of exploratory factor analysis: presentation, attitude, perception of food healthiness, and familiarity.

### 4.3. Correlation Matrix and Results of Hypothesis Testing

Table 3 shows the correlation matrix. Attitude positively correlates with the perception of food healthiness (r = 0.632, *p* < 0.05), presentation (r = 0.613, *p* < 0.05), and familiarity (r = 0.317, *p* < 0.05). The perception of food healthiness positively correlates with presentation (r = 0.639, *p* < 0.05) and familiarity (r = 0.343, *p* < 0.05). Presentation positively correlates with familiarity (r = 0.265, *p* < 0.05). The mean values of attitude, perception of food healthiness, presentation, and familiarity are 3.479, 3.033, 3.583, and 3.258, respectively.

Table 4 shows the results of hypothesis testing. Model 1 and Model 2 are statistically significant according to the F value (*p* < 0.05). Presentation positively affects both the perception of food healthiness (β = 0.296, *p* < 0.05) and attitude (β = 0.393, *p* < 0.05). The perception of food healthiness also positively impacts attitude (β = 0.453, *p* < 0.05). Familiarity × Presentation has a positive effect on attitude (β = 0.094, *p* < 0.05). The conditional effects of the focal predictor on familiarity increased in the following manners: 2.25 (β = 0.231, *p* < 0.05), 3.25 (β = 0.273, *p* < 0.05), and 4.25 (β = 0.316, *p* < 0.05). Therefore, all the proposed hypotheses are supported.

**Figure 2:**
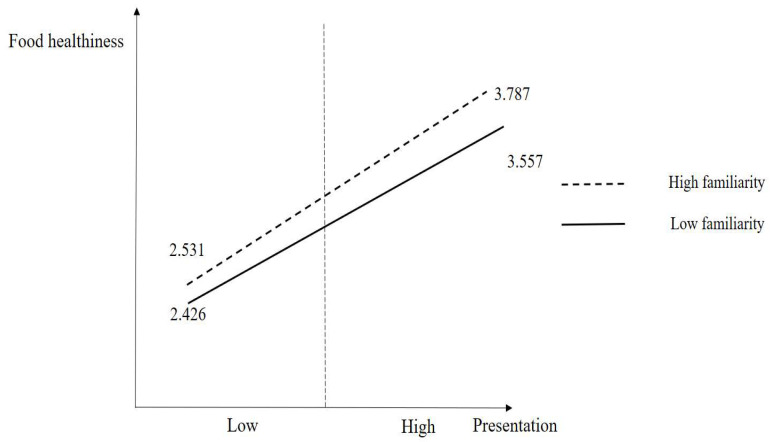
Results of the moderating effect of familiarity.

Figure 2 shows the results of the moderating effect of familiarity on the relationship between presentation and the perception of food healthiness. The four groups and mean values are presented in Figure 2 (low familiarity × low presentation: 2.426, high familiarity × low presentation: 2.531, low familiarity × high presentation: 3.557, and high familiarity × high presentation: 3.787).

## 5. Discussion and Conclusions

This research investigated the relationships among in-flight food presentation, perception of food healthiness, attitudes, and familiarity. This research was mainly based on an online survey to deal with consumer behavior in the domain of in-flight meals using convenience sampling. Convenience sampling is likely to become an adequate method for this work because the aim of this work is to inspect the general consumer perception focusing on in-flight meal consumption experiences. In addition, this research adopted random sampling because it could minimize the bias in the outcomes of the work [53]. Of the four attributes, survey participants were the most skeptical in their perception of food healthiness. This indicates that the food healthiness of in-flight meals is likely to become a point of weakness. However, survey participants appraised the presentation most positively out of the four attributes. This indicates that food presentation could become a strength in the area of in-flight meals. The results also revealed that presentation enhances the perception of food healthiness for in-flight meals. It can be inferred that food presentation is a very essential element in the domain of in-flight meals because nicely presented food led passengers to perceive the food as healthier. Moreover, the results revealed that food presentation plays a significant role in fostering a better attitude toward in-flight meals, and the positive impact of the perception of food healthiness on attitudes toward in-flight meals. It implied that offering nicely presented and nutritional food is critical to building better consumer attitudes. Furthermore, the results revealed a positive moderating effect of familiarity on the relationship between food presentation and the perception of food healthiness. It can be inferred that food presentation and familiarity could exert synergistic effects on establishing the perception of healthiness of in-flight meals. Indeed, the results showed that the increase in the perception of food healthiness in the high-familiarity group through improvement in food quality was greater than that in the low-familiarity group. Possibly, as in-flight meals might be consumed by both domestic and international passengers, providing customers with familiar food might be adequate for the purpose of establishing healthier perceptions of the food because passengers might not be able to know whether certain food is nutritional or not in case of unfamiliar food.

This research sheds light on the literature by identifying the relationships among four attributes. The results revealed a significant association among the four attributes. Additionally, this research found a significant moderating effect of familiarity on the relationship between food presentation and the perception of healthiness. Moreover, this research contributes to the literature by providing in-depth knowledge on in-flight meals. Although in-flight meals are critical in airline service, the extant literature has scantly examined the perception of food healthiness of in-flight meals. Thus, this study could contribute to the field by filling this research gap.

This work has practical implications. First, managers might be able to allocate more resources to make food presentation better. Because food presentation is determined by diverse attributes, such as color, freshness, and arrangement, airline managers might be able to improve the presentation of in-flight meals. Also, food presentation might need minimal additional costs from the perspective of managers, which might become a more efficient avenue to better performance. Moreover, perhaps airline managers could investigate how to provide healthy food to their customers. Because in-flight meals are consumed in uncomfortable situations that make passengers more exhausted, airline managers might need to focus on offering better nutrition to promote their health. Additionally, Plasek et al. [6] reported that food healthiness perception is influenced by various attributes: packaging, information, ingredients, sensory properties, and origin; these findings might serve as a reference for the marketing of in-flight meals. Additionally, airline managers might consider presenting familiar food to consumers. Because food familiarity includes cultural aspects, airline managers might need to identify their main customer demographics first. Such an effort might ultimately improve consumers’ attitudes toward in-flight meals, which could increase competitiveness for an airline that makes such an effort.

This research has limitations. First, this research depended on a survey based on the experience of the survey participants. Future research might consider an experimental design to study the perception of survey participants through offering stimuli such as photos and specific dishes to examine consumer behavior further. Moreover, this research considered only presentation as a determinant of food healthiness and attitude. Researchers could use more diverse attributes to explain both the perception of healthiness and attitudes toward in-flight meals. In addition, there was a greater proportion of female survey participants in this work. This might result in the results of this work being unbalanced. Future research might be able to consider more balanced data collection in case of gender to make more generalizable outcome.

## Figures and Tables

**Table 1:** Demographic information (*n* = 317).

Item	Frequency	Percentage
Male	61	19.2
Female	256	80.8
20s	80	25.2
30s	127	40.1
40s	83	26.2
50s	21	6.6
Older than 60	6	1.9
Monthly household income		
Under USD 2500	55	17.4
USD 2500 to 4999	93	29.3
USD 5000 to 7499	65	20.5
USD 7500 to 9999	36	11.4
Over USD 10,000	68	21.5
Annual usage frequency		
Less than 1 time	38	12.0
1~2 times	184	58.0
3~5 times	69	21.8
More than 5 times	26	8.2

**Table 2:** Results of exploratory factor analysis and reliability tests.

Attribute (Cronbach’s α)	Measurement Item	Loading	Eigen Value (VE(%))
Presentation (0.929)	The presentation of the in-flight meal was organized.	0.838	7.767 (48.541)
	The presentation of the in-flight meal looked good.	0.788	
	The presentation of the in-flight meal was neat.	0.869	
	The presentation of the in-flight meal was nice.	0.799	
Attitude (0.927)	For me, the in-flight meal was (negative–positive)	0.841	2.021 (12.630)
	For me, the in-flight meal was (bad–good)	0.833	
	For me, the in-flight meal was (unfavorable–favorable)	0.843	
	For me, the in-flight meal was (useless–useful)	0.745	
Food healthiness (0.903)	The in-flight meal was nutritious.	0.744	1.300 (8.123)
	The in-flight meal’s nutrition promoted my health condition.	0.822	
	The nutritional value of the in-flight meal was adequate.	0.720	
	The in-flight meal`s nutrition was excellent.	0.774	
Familiarity (0.765)	I was familiar with the in-flight meal.	0.819	1.163 (7.271)
	The in-flight meal was food that I frequently eat.	0.793	
	I was accustomed to the in-flight meal.	0.823	
	The in-flight meal was not unfamiliar with me.	0.566	

Note: Kaiser–Meyer–Olkin (KMO) measure of sampling adequacy = 0.916, Bartlett’s test of sphericity χ^2^ = 3935.788, total variance explained: 76.565%, VE stands for variance explained.

**Table 3:** Correlation matrix.

Variable	Mean	SD	1	2	3	4
1. Attitude	3.479	1.049	1			
2. Food healthiness	3.033	0.942	0.632 *	1		
3. Presentation	3.583	0.940	0.613 *	0.639 *	1	
4. Familiarity	3.258	0.974	0.317 *	0.343 *	0.265 *	1

Note: * *p* < 0.05, SD stands for standard deviation.

**Table 4:** Results of hypothesis testing.

	Model 1 Food Healthiness		Model 2 Attitude	
	β	t Value	β	t Value
Constant	1.352	2.93 *	0.695	3.97 *
Presentation	0.296	2.29 *	0.393	6.61 *
Familiarity	−0.156	−1.07		
Interaction	0.094	2.41 *		
Food healthiness			0.453	7.64 *
F value	85.80 *		141.04 *	
R^2^	0.4513		0.4732	
Conditional effect of focal predictor				
Familiarity				
2.25	0.231	9.28 *		
3.25	0.273	13.77 *		
4.25	0.316	11.20 *		
Index of mediated moderation	Index	LLCI	ULCI	
	0.0429 *	0.0042	0.0853	

Note: * *p* < 0.05, Interaction: Familiarity × Presentation (test of unconditional interaction: F = 5.85 *).

## Data Availability

The data presented in this study are available on request from the corresponding author due to privacy.
